# Comparing neural substrates of emotional vs. non-emotional conflict modulation by global control context

**DOI:** 10.3389/fnhum.2014.00066

**Published:** 2014-02-13

**Authors:** Maryem Torres-Quesada, Franziska M. Korb, Maria J. Funes, Juan Lupiáñez, Tobias Egner

**Affiliations:** ^1^Department of Experimental Psychology, Mind, Brain and Behavior Research Center, Universidad de GranadaGranada, Spain; ^2^Department of Psychology and Neuroscience, Center for Cognitive Neuroscience, Duke UniversityDurham, NC, USA

**Keywords:** cognitive control, control context, congruency sequence effects, proportion congruent effects, emotional conflict, non-emotional conflict, fMRI

## Abstract

The efficiency with which the brain resolves conflict in information processing is determined by contextual factors that modulate internal control states, such as the recent (local) and longer-term (global) occurrence of conflict. Local “control context” effects can be observed in trial-by-trial adjustments to conflict (congruency sequence effects: less interference following incongruent trials), whereas global control context effects are reflected in adjustments to the frequency of conflict encountered over longer sequences of trials (“proportion congruent effects”: less interference when incongruent trials are frequent). Previous neuroimaging and lesion studies suggest that the modulation of conflict-control processes by local control context relies on partly dissociable neural circuits for cognitive (non-emotional) vs. emotional conflicts. By contrast, emotional and non-emotional conflict-control processes have not been contrasted with respect to their modulation by global control context. We addressed this aim in a functional magnetic resonance imaging (fMRI) study that varied the proportion of congruent trials in emotional vs. non-emotional conflict tasks across blocks. We observed domain-general conflict-related signals in the dorsal anterior cingulate cortex (dACC) and pre-supplementary motor area and, more importantly, task-domain also interacted with global control context effects: specifically, the dorsal striatum and anterior insula tracked control-modulated conflict effects exclusively in the emotional domain. These results suggest that, similar to the neural mechanisms of local control context effects, there are both overlapping as well as distinct neural substrates involved in the modulation of emotional and non-emotional conflict-control by global control context.

## Introduction

Cognitive control refers to processes that guide perceptual and motor selection in line with task goals, especially in the face of distraction from irrelevant stimuli or task-inappropriate response tendencies (Miller and Cohen, 2001). In many contexts goal-driven behavior requires responses that are based on the selection of relevant sources of information amidst competing sources of distraction, like successfully navigating one's way through crowded traffic while ignoring the car radio and colorful advertising boards. In the laboratory, this type of goal-driven attentional selection is exemplified by the classic color-naming Stroop task (for a review see Macleod, 1991), where participants are required to name the ink color in which color-words are displayed, and the meaning of the words can be congruent or incongruent with their ink color. Participants need to select the relevant information (the ink color) over the irrelevant information (word meaning) to perform successfully, which is rendered particularly difficult by the fact that word-reading is a highly practiced process whereas color-naming is not. Therefore, response times (RTs) are reliably slower for trials where the meaning of the word stimulus is incongruent with its color (e.g., the word RED printed in green) compared to trials where the word and color are congruent (e.g., the word RED printed in red). The difference in performance between incongruent and congruent trials is called the congruency or conflict effect, and is used as an index of the relative success (or failure) to impose cognitive control and selectively process task-relevant vs. -irrelevant information.

Various task parameters have been found to modulate conflict effects; in particular, the local (short-term) and global (longer-term) “control context,” as defined by the incidence of congruent vs. incongruent stimuli, is known to affect the size of the congruency effect. Local control context effects are reflected in the modulation of congruency effects by (first-order) trial-by-trial congruency transitions (often called “congruency sequence” or “conflict adaptation” effects, for a review see Egner, 2007), whereas global control context effects relate to the modulation of the congruency effect by the frequency of congruent relative to incongruent stimuli over longer sequences of trials (“proportion congruent” effects, for a recent review, see Bugg and Crump, 2012). If certain lower-level feature repetition and stimulus-response learning effects are accounted for (Mayr et al., 2003; Hommel et al., 2004; Schmidt and Besner, 2008), both of these modulations are typically considered reflections of control processes.

Specifically, local control context (congruency sequence) effects are defined by conflict effects that are smaller on a current trial when preceded by an incongruent trial than by a congruent trial (Gratton et al., 1992). This phenomenon has been interpreted to reflect a transient or reactive conflict-control process, where conflict generated during an incongruent trial leads to a compensatory up-regulation in top–down control that is observed in the form of reduced congruency effects on the following trial (Egner, 2007; Egner et al., 2010). On the other hand, global control context (proportion congruent) effects are measured by manipulating the relative proportions of congruent and incongruent trials within an experimental block. The magnitude of the congruency effect varies with the proportion of congruent trials, being larger in the context of a high proportion of congruent trials than in the context of a low proportion of congruent trials (e.g., Logan and Zbrodoff, 1979; Lowe and Mitterer, 1982; West and Baylis, 1998; Carter et al., 2000). This effects is typically attributed to a strategic adoption of a higher level of sustained or proactive top–down control in response to encountering frequent conflict (i.e., when the proportion of incongruent trials is high) and a relaxation of control when conflict is rare (i.e., when the proportion of congruent trials is high) (Carter et al., 2000; Krug and Carter, 2012). Interestingly, several behavioral studies suggest that adjustments to conflict-control processes driven by local vs. global control context are driven by dissociable mechanisms, as these effects differ with respect to their conflict-specificity (Funes et al., 2010) and global effects can be observed in the absence of local ones (Torres-Quesada et al., 2013).

Congruency sequence effects and their neural correlates have been extensively investigated (e.g., Botvinick et al., 1999, 2001; Durston et al., 2003; Kerns et al., 2004; Egner and Hirsch, 2005a,b), with much evidence suggesting key roles for the dorsal anterior cingulate cortex (dACC) and the dorsolateral prefrontal cortex (dlPFC). Moreover, a number of studies have provided strong evidence for a distinction between circuits involved in detecting and resolving cognitive (non-emotional) conflict from those that detect and resolve emotional conflict (Etkin et al., 2006; Mohanty et al., 2007; Egner et al., 2008; Monti et al., 2010; Maier and di Pellegrino, 2012). Specifically, whereas in non-emotional contexts, conflict appears to be detected in the dACC (Botvinick et al., 1999; Kerns et al., 2004) and subsequent control adjustments implemented by the dlPFC (Kerns et al., 2004; Egner and Hirsch, 2005a,b) through biasing of stimulus processing in posterior sensory regions (Egner and Hirsch, 2005b), emotional conflict detection appears to also involve the dACC (and additionally the amygdala), but subsequent control adjustments have been mapped onto the pregenual, rostral ACC (rACC) inhibiting amygdala activation (Etkin et al., 2006, 2010; Egner et al., 2008; Krug and Carter, 2010; Maier and di Pellegrino, 2012).

Compared to congruency sequence effects, the neural correlates of global control context (proportion congruent) effects have been studied less extensively (Carter et al., 2000; Grandjean et al., 2012; Krug and Carter, 2012; Wilk et al., 2012). Given that this effect is assessed at the level of blocks of trials, neural signatures can be investigated both for a putative sustained control process (which would be more engaged in low than in high proportion congruent blocks) as well as for phasic (event-based) conflict signals (i.e., the contrast between incongruent and congruent trials) as a function of control context (block membership). In an early study, Carter et al. (2000) focused on the latter, and showed the ACC to track transient conflict signals to incongruent stimuli as modulated by the proportion of congruent trials (i.e., conflict signals were less pronounced under low than high proportion congruent contexts). More recent studies attempted to tease apart sustained and transient (stimulus-evoked) neural signatures in a single protocol. One study reported fronto-parietal activity, including dlPFC and ACC, tracking phasic conflict signals as a function of global control contexts, but no sustained activity varying across the different proportion congruent contexts (Grandjean et al., 2012), whereas another study found sustained control signals (low > high proportion congruent conditions) in the medial frontal cortex and phasic context-modulated conflict signals in the ACC, inferior frontal junction and anterior insula (Wilk et al., 2012).

Moreover, Krug and Carter (2012) investigated proportion congruent effects in the context of emotional conflict and also found medial and lateral PFC activation (plus right amygdala) to track phasic conflict signals modulated by global control context, but they additionally reported (based on a separate model assessing only block-wise activation) higher sustained responses for low than high proportion congruent blocks in the right dlPFC, suggesting an up-regulation of activity in this region in contexts where incongruent trials are frequent. The latter study raises the intriguing possibility that, akin to the distinction between non-emotional and emotional neural mechanisms involved in the modulation of conflict processing by local control context, there might also be distinct, domain-specific neural mechanisms involved in the modulation of conflict processing by global control context. However, since that study consisted only of an emotional conflict task without a comparison condition of non-emotional conflict, it remains unknown whether the modulation of emotional conflict by proportion congruency relies on distinct neural substrates from those of non-emotional conflict.

The main goal of the present study then was to investigate whether there are dissociable neural mechanisms involved in the modulation of emotional vs. non-emotional conflict by global control context, by providing an appropriate comparison condition. To this end, participants performed two face-word Stroop tasks (cf. Egner et al., 2008)—one using emotional stimuli and the other one non-emotional stimuli—while brain activity was recorded using functional magnetic resonance imaging (fMRI). The proportion of congruency was manipulated between blocks within each task, alternating between high and low proportion congruent conditions. Note that this design was not optimized for a “hybrid” blocked/event-related assessment of simultaneous sustained and event-related responses (Dosenbach et al., 2006; Petersen and Dubis, 2012). Rather, we focused on event-related conflict signals (i.e., current trial congruency effects) in emotional and non-emotional task domains, and their modulation by global control context. Additionally, we could also assess the modulation of conflict responses by local control context (as defined by the congruency of the preceding trial).

We chose this approach for two main reasons: first, a frequency-based design like the proportion congruent manipulation inherently entails a strong dependence between block and event types (e.g., a high global control context is defined by frequent incongruent trials); this non-orthogonality is obviously suboptimal for assessing putatively independent contributions of sustained and phasic fMRI responses, which can be easily misattributed in hybrid analyses even when the underlying blocked- and event-based factors are orthogonal by design (e.g., Visscher et al., 2003). Second, in extensive behavioral piloting, we observed reliable proportion congruent effects only when employing blocks of trials of much longer duration than those known to be optimal for assessing block-related fMRI responses (e.g., Wager and Nichols, 2003). Thus, the advantage of the present approach is that we could produce the basic behavioral phenomena of interest and we do not run the risk of misattributing event-related to sustained responses (and *vice versa*), but at the cost of foregoing a “hybrid” blocked/event-related analysis that could, in theory, gauge independent phasic and sustained neural responses.

Specifically, we aimed to identify regions involved in *general* conflict processing (displaying greater activity for incongruent than congruent trials, irrespective of other factors), regions involved in conflict processing as modulated by global control context (by assessing congruency effects as a function of the proportion congruent manipulation), and regions involved in conflict processing as modulated by local control context (by assessing current trial congruency effects as a function of previous trial congruency). Most importantly, we searched for brain areas that displayed these effects in a domain-specific fashion, by analyzing the interaction between the above contrasts with the factor of task-domain (non-emotional vs. emotional). Behaviorally, we expected to observe comparable congruency, proportion congruent, and congruency sequence effects in both task-domains. At the neural level, we expected to replicate the previous findings on basic conflict and local control context effects (and their interaction with task-domain) that were described above (e.g., Egner and Hirsch, 2005a,b; Etkin et al., 2006; Egner et al., 2008). For the analyses involving these effects' interactions with the global control context factor, we had less specific expectations, since comparable contrasts had not been carried out in the prior literature. The key question was whether any one region, or network of regions, would be selectively affected by global control context as a function of task-domain, thus providing evidence for dissociable neural substrates of global control context modulation in emotional vs. non-emotional task settings.

## Methods

### Overview

The study consisted of two phases, a main task and a subsequent independent localizer task. The main task served the assessment of the neural substrates of global and local control context effects in emotional vs. non-emotional domains, as described in the Introduction. The subsequent localizer task served to independently identify face-sensitive brain regions in the fusiform gyrus (the fusiform face area, FFA) (Kanwisher et al., 1997) and in the amygdala. These regions could then serve as independently defined regions-of-interest (ROIs) in interrogating the effects of global and local control contexts assessed in the main task data-set, supplementing the standard whole-brain analyses. The FFA and amygdala were of special interest due to their well-known involvement in face processing (FFA and amygdala) and emotional processing (amygdala).

### Participants

Twenty-four right-handed volunteers gave written informed consent to participate in this study, which was approved by the Duke University Health System Institutional Review Board. All participants had normal or corrected-to-normal vision, and reported no current or history of neurological, psychiatric, or major medical disorder. They were reimbursed with $30 for their participation, which lasted approximately 90 min. The data of three participants were excluded due to incomplete scans (two participants) or high error percentage (one participant with >30% errors). The remaining twenty-one participants were 10 females and 11 males (mean age = 24.8; range = 19–34).

### Stimuli

**Figure 1:**
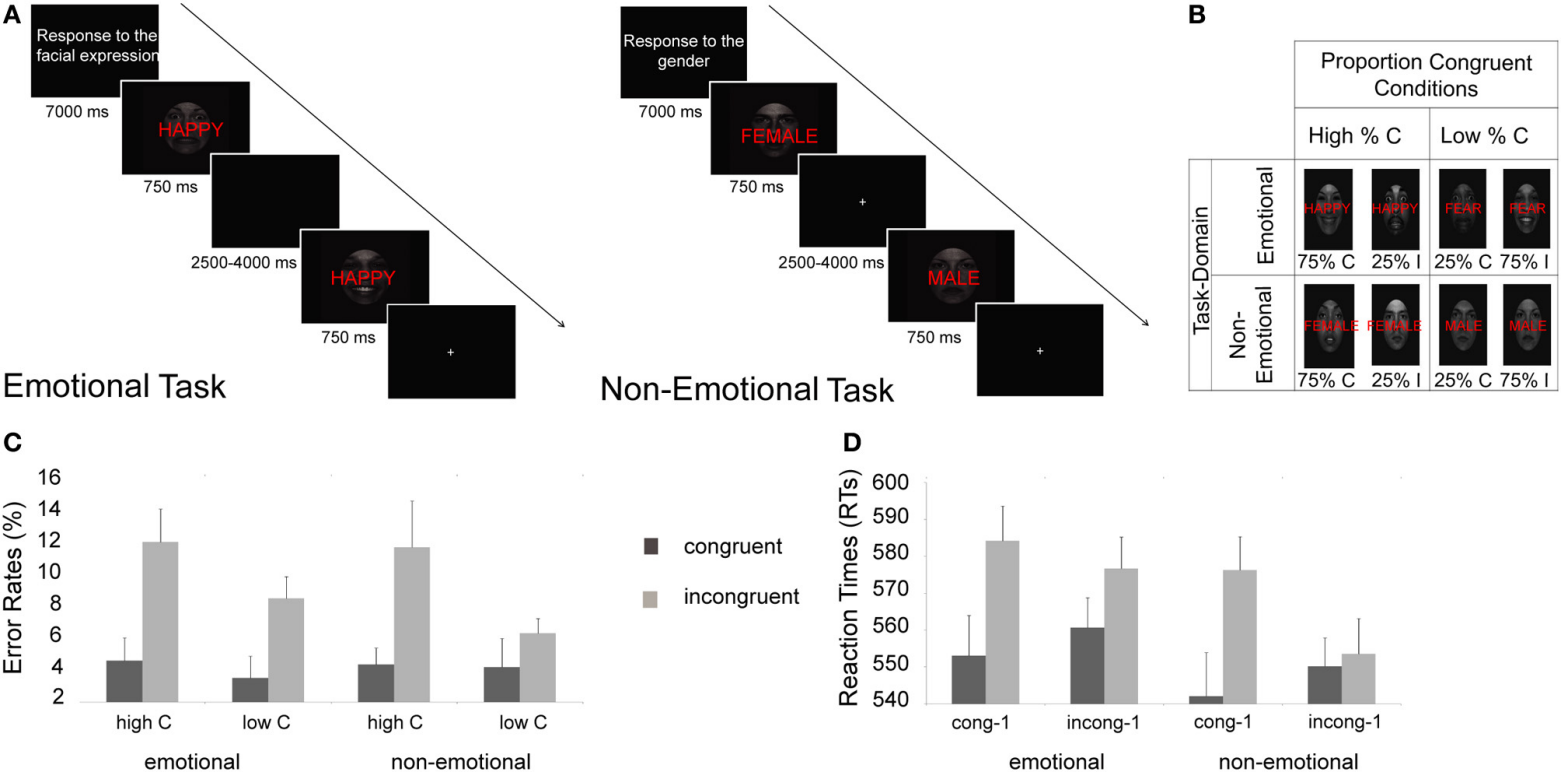
**Experimental protocol, design, and behavioral results. (A)** Instructions and two example trials are shown for the emotional (left panel) and non-emotional (right panel) tasks. **(B)** Manipulation of proportion congruent trials for each task-domain condition (C, congruent; I, incongruent). **(C)** Mean error rates (+s.e.m.) display the classic global control context (proportion congruent) effect, that is, differences in error rates between congruent and incongruent trials are smaller in the low proportion congruent (“low C”) than in the high proportion congruent (“high C”) condition, in both task-domains. **(D)** Mean reaction times (+s.e.m.) display the classic local control context (congruency sequence) effect, that is, differences in RT between congruent and incongruent trials are smaller when the previous trial was incongruent (“incong-1”) than when the previous trial was congruent (“cong-1”).

Stimuli were displayed on a back-projection screen that was viewed by participants via a mirror attached to the head-coil. This set-up simulated a viewing distance of approximately 80 cm, resulting in individual stimuli extending ~9° horizontally and ~11° vertically for the face-word task and ~10° horizontally and ~12° vertically for the localizer. For the face-word task, stimuli were presented using E-prime software (Psychology Software Tools, Pittsburgh, PA), and consisted of photographic gray-scale images displayed on a black background, depicting male or female faces posing either happy, fearful, or neutral emotional expressions (NimStim faces database, Tottenham et al., 2009). Face stimuli were cropped to remove any hair. The stimulus set consisted of 24 unique images, 12 males (3 happy, 3 fearful, and 6 neutral faces) and 12 females (3 happy, 3 fearful, and 6 neutral faces). Each stimulus was presented with a red-capital distracter word overlaid on the face (Figure 1). The word could be “MALE,” “FEMALE,” “HAPPY,” or “FEAR.” For the localizer task, gray-scale pictures of faces or houses were presented on a gray background screen using MATLAB software (Mathworks Inc., Nantucket, MA). Face and house pictures for the localizer were obtained from an in-house collection.

### Procedure

Participants performed two tasks during fMRI: a face-word interference task (including an emotional and a non-emotional version), which was performed first, and a subsequent standard localizer task to provide independent functional definitions of the FFA and the amygdala as ROIs. The face-word task consisted of two blocks of 16 practice trials each (not included in the statistical analysis and performed outside the scanner), followed by 4 runs of the experimental task (each run comprised 2 blocks of 64 trials each, resulting in a total of 512 trials). The first two runs were of one block type (e.g., emotional blocks) and the other two runs were of the other type (e.g., non-emotional blocks), and this order was counterbalanced across participants.

For the emotional blocks, 12 emotional faces were presented, 6 of them showing happy faces (3 males and 3 females) and the other 6 showing fearful faces (3 males and 3 females). For the non-emotional blocks, all the faces (6 males and 6 females) had neutral facial expressions. Thus, each task involved 12 unique facial identities, such that the number of exposures (and familiarity) with the face stimuli was equated across the two task-domains. Like the faces, distracters were grouped by blocks: in emotional block, only happy and fear words were displayed, while in non-emotional blocks, only female and male words were presented. Stimuli were presented in a random order within each block, with the constraint that a given face image was never repeated across consecutive trials, in order to avoid potential confounds from repetition priming or low-level feature integration effects (Mayr et al., 2003; Hommel et al., 2004).

Participants were instructed to categorize the face stimuli while ignoring the word distracters. Specifically, they had to indicate whether the face was happy or fearful in the emotional task blocks, or whether the face was male or female in the non-emotional task blocks. Given the possible pairings between target face stimuli and distracter word labels, these tasks produced congruent and incongruent stimuli, akin to the classic Stroop task (Egner et al., 2008). Specifically, the congruency factor arises from a match or mismatch between face and word stimuli, that is, when both indicate the same response (i.e., happy facial expression with a happy overlaid word) the trial is *congruent*, whereas when they do not (i.e., happy facial expression with a fear overlaid word) the trial is *incongruent*. Besides this congruency factor, the proportion of congruent and incongruent trials within block was manipulated, presenting 75% of congruent trials and 25% of incongruent for the *high proportion congruent condition* (corresponding to a “low” global control context), and 75% of incongruent trials and 25% of congruent for the *low proportion congruent condition* (corresponding to a “high” global control context). Moreover, proportion of congruency alternated across the 8 blocks, starting with the low proportion congruent condition (i.e., low, high, low, and so on); this order of blocks was found to be most effective in producing robust proportion congruent effects in behavioral pilot work, as has been recently reported (Abrahamse et al., 2013).

Participants responded to the stimuli using their right hand index and middle fingers to press buttons on a MRI-compatible response box, which was vertically oriented on the participant's chest. Stimulus-response mappings were counterbalanced across participants. Since both the non-emotional and emotional tasks have the same response mappings, the associations between their factor levels (e.g., happy and female faces being responded with the same key) were also counterbalanced across participants.

Stimuli were presented for 750 ms, followed by a jittered inter-trial interval (ITI) during which a fixation cross was displayed centrally on the screen. To facilitate optimal statistical segregation of blood-oxygenation-level-dependent (BOLD) signals across successive trials, the ITI was randomly drawn from a pseudo-exponential distribution, where 50% of interval lasted 2.5 s, 25% lasted 3 s, 16% lasted 3.5 s, and 9% lasted 4 s (mean interval ~3 s). At the beginning of each block, instructions indicating whether the subjects had to respond to the gender or to the expression of the faces were displayed for 7 s, and there were 3 s intervals between blocks within each run.

The face-network localizer task consisted of a 1-back task, where participants were required to push the right hand index finger response button whenever two identical stimuli were presented in a row. Twelve blocks of 15 stimuli each were presented, alternating between blocks where only faces were displayed and blocks where only houses were presented. Each stimulus appeared on the screen for 750 ms, separated by a 250 ms ITI, and a 10 s fixation period between blocks. The purpose of this standard localizer scan is to define face-sensitive regions of the FFA and amygdala (via a face-blocks > house-blocks contrast) that could then be employed as independently defined ROIs to supplement the whole-brain analyses of the main task.

### Design

As outlined above, there were two types of task-domains used in the main experiment (emotional vs. non-emotional), a congruency factor with two levels (congruent vs. incongruent), and two proportion congruent conditions (low vs. high proportion congruent, corresponding to high vs. low global control contexts). In addition to these variables, we coded sequential effects offline by creating one additional within-subjects variable, *previous trial congruency*, representing the level of congruency encountered on the previous trial (congruent vs. incongruent, corresponding to low vs. high local control contexts). The factorial combination of these 4 factors formed our 16 experimental conditions.

### Image acquisition

Images were recorded on a 3T GE Signa EXCITE HD system using a standard 8-channel birdcage head coil. Functional images were acquired parallel to the anterior-posterior commissure line with a T2^*^-weighted single-shot gradient EPI sequence of 30 contiguous axial slices [time repetition (*TR*) = 2000 ms, time echo (*TE*) = 28 ms, flip angle = 90°, field of view = 192 mm, array size 64 × 64] with 3.5 mm thickness and 3 × 3 mm in plane-resolution. Structural images were acquired with a T1-weighted SPGR sequence using a 3D inversion recovery prepared sequence, recording 180 slices of 1 mm thickness in plane-resolution of 1 × 1 mm (*TR* = 7.48 ms, *TE* = 2.98 ms, field of view = 256 × 256 mm).

### Image preprocessing

All preprocessing and statistical analyses were carried out using SPM8 (http://www.fil.ion.ucl.ac.uk/spm/software/spm8/). Functional data were slice-time corrected and spatially realigned to the first volume of the first run. The structural scan was co-registered to the functional images, and served to calculate transformation parameters for spatially warping functional images to the Montreal Neurological Institute (MNI) template brain (resampled voxel size: 2 mm^3^). Finally, normalized functional images were spatially smoothed with an 8 mm^3^ full-width-half-maximum Gaussian kernel. The first 5 volumes of each run were discarded prior to building and estimating the statistical models. In order to remove low-frequency confounds, data were high-pass filtered (128 s). Temporal autocorrelations were estimated using restricted maximum likelihood estimates of variance components using a first-order autoregressive model (AR-1), and the resulting non-sphericity was used to form maximum likelihood estimates of the activations.

### Image analyses

Regressors for stimulus events (convolved with a canonical hemodynamic response function) were created for each of the combinations of task-domain (emotional vs. non-emotional), proportion congruent (low proportion vs. high proportion), previous trial congruency (incongruent vs. congruent) and current trial congruency (congruent vs. incongruent) factors, resulting in a total of 16 different trial types/regressors. Additionally, we modeled error trials, the first trial of each block, and instruction screens as nuisance regressors. This model was applied to each subject's data, followed by linear contrasts between events of interest. Specifically, we computed the main effect of congruency (*general conflict processing*), the interaction between congruency and proportion congruency (*global control context*), the interaction between previous trial and current trial congruency (*local control context*), as well as the interactions between these contrasts and the task-domain factor (emotional vs. non-emotional). Group effects were assessed by submitting the individual SPMs for the above contrasts to voxel-wise *t*-tests at the group level, where subjects were treated as random effects.

Note that the nature of the proportion congruent manipulation necessarily results in relatively low trial counts for the “low proportion” conditions. This issue is further compounded when splitting up trial types over multiple additional factors. We therefore chose to pursue only the above-mentioned 2- and 3-way interaction analyses, where the trial count for the smallest cells in the 3-way interaction analyses was 32 trials (for 2-way interactions, the smallest cells had 64 trials). By contrast, we did not pursue an analysis of 4-way interaction effects between congruency, global control context, local control context, and task domain, because this analysis would involve some cell sizes of only 16 trials, which may render unreliable results. Moreover, it should be emphasized that our models assess exclusively event-related activity rather than pursuing a hybrid blocked/event-related approach, where sustained (block-wise) activation is modeled in addition to event-related responses (e.g., Dosenbach et al., 2006). The reason we opted against the latter is that our design was not optimized for minimizing colinearity between block- and event-based effects. Similarly, our task blocks were rather long (~300 s), which renders the modeling of sustained activation sub-optimal (Wager and Nichols, 2003). We employed such long blocks based on extensive behavioral piloting, where robust PC effects were observed only for relatively long blocks.

To control for false-positive rates in analyzing the main task data, combined voxel activation intensity and cluster extent thresholds corrected for multiple comparisons were determined by using 3dClustSim of the AFNI software suite (http://afni.nimh.nih.gov/afni/). Specifically, the program was used to run 10,000 Monte Carlo simulation taking into account the whole-brain search volume and the estimated smoothness of the residuals of the respective group SPMs to generate probability estimates of a random field of noise producing a cluster of voxels of a given extent for a set of voxels passing a specific voxel-wise *p*-value threshold, which we set at *p* < 0.005 for all analyses. Given this voxel-wise threshold, the simulations determined that cluster sizes ranging between 162 and 261 voxels, depending on the specific group contrast, corresponded to a combined threshold of *p* < 0.05 (whole-brain corrected). Following identification of activations that passed the whole-brain corrected thresholds for interaction effects, we followed up these analyses by extracting mean cluster activation values using Marsbar software (http://marsbar.sourceforge.net/) in order to determine the likely causes for each interaction effect (and to display the data patterns in graphical form). All reported *t*-tests are 2-tailed.

Finally, we also created functionally defined FFA and amygdala ROIs from the independent localizer task and used these to extract activation estimates for these regions during the main task. Given that the localizer data were independent of the main task and only served to define face-sensitive voxels in the FFA and amygdala, we employed a more lenient, arbitrary statistical threshold (voxel-wise *p* < 0.005, cluster extent = 20) in defining activations in these regions (for FFA group ROI, see Figure 4A). Since face-related activation in the amygdala was quite extensive at this threshold, with activation clusters extending beyond the anatomical borders of this region, the functional amygdala ROI was furthermore masked with an anatomically defined amygdala ROI taken from the WFU pickatlas (http://fmri.wfubmc.edu/software/PickAtlas).

## Results

### Behavioral data

Descriptive statistics for RT and error rate performance measures for each experimental condition are presented in Table 1. For the analysis of mean RTs, we excluded the first trial of each block and error trials. To reduce the influence of outlier values on the RT analyses, data were trimmed using *a priori* fixed cut-offs aimed at excluding fast guesses (*RT* < 150 ms) and very slow (presumably inattentive) responses (*RT* > 1500 ms), resulting in an exclusion of 9.4% of all trials. While these trimming values are arbitrary, they lie within the conventional range of cut-offs used in RT distributions of comparable 2-alternative forced-choice tasks, where fixed cut-offs have been shown to be an effective (and simple) methods for increasing power (Ratcliff, 1993). For the analysis of error rates only the first trial of each block was eliminated. One repeated-measures ANOVA was carried out on each dependent variable (mean RTs and error rates), including task-domain (emotional vs. non-emotional), global control context (low vs. high proportion of congruent trials), local control context (previous trial congruency: congruent vs. incongruent), and current trial congruency (congruent vs. incongruent) as within-participant factors.

**Table 1:** **Descriptive statistics of behavioral data**.

	**High% C**				**Low% C**			
	**C-1**		**I-1**		**C-1**		**I-1**	
	**C**	**I**	**C**	**I**	**C**	**I**	**C**	**I**
Emotional	541 (59)	569 (46)	559 (51)	567 (42)	565 (48)	599 (59)	562 (52)	586 (70)
	4.82 (5.95)	15.01 (8.69)	4.41(4.84)	9.17(11.47)	1.63 (5.23)	8.41(8.42)	5.45 (6.05)	8.71 (6.63)
Non-emotinal	537 (81)	571 (77)	554 (71)	566 (73)	547 (84)	581 (75)	546 (70)	541 (69)
	4.51 (3.73)	11.66 (10.5)	4.27 (4.40)	11.88 (17)	3.17 (11.33)	7.30 (5.79)	5.24(5.52)	5.40(5.37)

Upper rows denote RT (SD) in ms, lower rows denote percentage (SD) of error trials. C, Congruent; I, Incongruent; C-1, Previous Congruent; I-1, Previous Incongruent; High% C, High proportion of congruent trials; Low% C, low proportion of congruent trials.

The RT data displayed a main effect of congruency, *F*_(1, 20)_ = 24.29, *p* < 0.001, with slower RTs for incongruent trials (573 ms) compared to congruent ones (551 ms). The effect of congruency was furthermore modulated by local control context, *F*_(1, 20)_ = 12.67, *p* < 0.005, showing the typical congruency sequence effect, that is, larger congruency effects when the previous trial was congruent [*F*_(1, 20)_ = 45.39, *p* < 0.001, 33 ms congruency effect] than when the previous trial was incongruent [*F*_(1, 20)_ = 2.75, *p* > 0.1, 10 ms congruency effect]. This interaction was not modulated by task [*F*_(1, 20)_ = 1.79, *p* > 0.1]. We also detected a marginal interaction between task-domain, global control context, and congruency, *F*_(1, 20)_ = 3.28, *p* = 0.085, due to a numerically larger global control context effect for the non-emotional task (that is, larger congruency effects for high proportion of congruent trials condition, 23 ms, than for the low proportion of congruent trials condition, 15 ms) than for the emotional task where no global control context effect on RT was detected (18 and 29 ms for high and low proportion of congruent trials condition, respectively). The interaction between proportion congruent, previous congruency and congruency factors was not significant (*F* < 1), and neither was the 4-way interaction involving task-domain [*F*_(1, 20)_ = 1.31, *p* = 0.266] (see Figure 1D).

For error rates, we also observed a main effect of congruency, *F*_(1, 20)_ = 25.34, *p* < 0.001, with higher error rates for incongruent (10%) than for congruent trials (4%). The congruency effect was modulated both by local [*F*_(1, 20)_ = 4.54, *p* < 0.05] and global control context [*F*_(1, 20)_ = 7.09, *p* < 0.05], showing the typical pattern of congruency sequence and proportion congruent effects (Figure 1C). For local control context effects, congruency effects following a congruent trial were larger [7%, *F*_(1, 20)_ = 30.13, *p* > 0.001] than those following an incongruent trial [4%, *F*_(1, 20)_ = 8.62, *p* < 0.01]. Similarly, for global control context effects, smaller congruency effects were found in the low proportion congruent condition [4%, *F*_(1, 20)_ = 13.79, *p* < 0.005] than in the high proportion congruent condition [7%, *F*_(1, 20)_ = 22, *p* < 0.001]. Task-domain did not interact with either of these effects [*F*_(1, 20)_ = 1.08, *p* > 0.1 for congruency sequence effects, and *F* < 1 for proportion congruent effects]. Akin to the reaction time data, the interaction between global and local control context effects (proportion congruent × previous trial congruency × congruency) was not significant (*F* < 1), and neither was the 4-way interaction involving task domain [*F*_(1, 20)_ = 1.78, *p* = 0.20].

As noted in the Methods section, we avoided repetition of face stimuli over successive trial in order to preclude low-level feature priming effects from obscuring the assessment of local control context effects (Mayr et al., 2003; Hommel et al., 2004). However, concerns have also been raised about the degree to which global control processes are implicated in proportion congruent effects (Bugg et al., 2008; Schmidt and Besner, 2008). First, proportion congruent effects can at times be entirely accounted for by “item-specific” control effects, where in the low proportion congruent conditions a specific stimulus (e.g., a particular face in the present study) may come to “prime” a heightened control state due to its repeated pairing with incongruent distracters (Bugg et al., 2008). However, this possibility is rendered highly unlikely in the present study due to the inclusion of a large number of distinct face stimuli (12) (Bugg and Hutchison, 2012).

Another process that could theoretically drive proportion congruent effects relates to subjects learning to link the distracter labels to the correct responses (e.g., Schmidt and Besner, 2008). Here, the assumption is that subjects may in high proportion congruent blocks start to respond to the word labels (rather than the faces), and similarly, in the low proportion congruent blocks they may use the word labels to guide their responses by internally “flipping” the instructed gender- and emotion-response mappings (or “assigning a negative weight” to the label meaning, see Logan and Zbrodoff, 1979). If subjects employed these distracter-based response strategies in the present study, we would expect to obtain the following data patterns: first, one would expect to observe rather high error rates, because the distracter labels would indicate the incorrect response on 25% of the trials. However, we observed low error rates (mean = 6.7%). Second, if subjects based their responses on the word labels, the two “high contingency conditions” (congruent trials in high proportion congruent blocks and incongruent trials in low proportion congruent blocks) should produce similar RTs and error rates. Instead, the behavioral data are markedly different between these conditions (see Table 1). Finally, if subjects were to mentally switch the gender-to-response mapping in low proportion congruent blocks, this would effectively reverse the congruency of the stimuli, which should result in inverted congruency effects (see Logan and Zbrodoff, 1979). Again, this was not the case in our data set. Thus, we consider it highly unlikely that the present proportion congruent effects could be attributed to contingency learning effects.

In sum, we observed typical and reliable congruency and congruency sequence (local control context) effects in both the RT and accuracy data across task domains. Moreover, we observed proportion congruent (global control context) effects in the accuracy data, but not in RT data, for both task domains. Global and local control context effects did not interact with each other (see also Torres-Quesada et al., 2013). These behavioral results document that the experimental manipulations were effective in producing modulation of conflict processing by local and global control context, which sets the stage for interrogating the fMRI data for the neural substrates of these effects.

### fMRI data

The aim of the fMRI analyses was to reveal brain regions involved in either domain-general or domain-specific conflict-control processes. Specifically, we sought to identify regions involved in conflict processing (greater event-related signals for incongruent than congruent trials), and regions where these responses were modulated by global control context (where congruency effects are modulated by the proportion congruent manipulation) and local control context (where congruency effects are modulated by previous trial congruency). Most importantly, we additionally searched for brain areas that displayed these activation profiles in a domain-specific fashion, by analyzing interactions of the above contrasts with task-domain (non-emotional vs. emotional). All reported activations passed whole-brain correction (*p* < 0.05) via a combined voxel-height and cluster-extent threshold (see Methods) and are listed in Table 2. For any significant interaction effect results, mean cluster activation estimates were extracted (see Methods) and submitted to follow-up tests to determine the source of the interaction. In the case where clusters were so large that they spanned more than one anatomical area, these follow-up analyses were conducted both for the mean activity values across the entire cluster as well as for 5 mm spherical ROIs centered on the highest cluster peak within each anatomical region covered by the cluster.

**Table 2:** **Summary of fMRI Results**.

**Anatomical area**	**Hemisphere**	***x***	***y***	***z***	**Extent**	***Z*_max_**
**MAIN EFFECT OF CONFLICT (INCONGRUENT > CONGRUENT)**						
dACC/preSMA	L/R	8	18	40	900	3.61
Inferior frontal gyrus	R	46	14	−4	607	4.23
Inferior frontal gyrus/precentralgyrus	R	38	4	36	512	4.02
Middle temporal gyrus	R	50	−34	−2	253	3.40
**TASK × CONGRUENCY INTERACTION**						
Middle occipital and fusiform gyri	R	30	−86	8	491	3.81
**TASK × PROPORTION CONGRUENT × CONGRUENCY INTERACTION**						
Dorsal striatum/thalamus	L	−28	−2	6	1683	4.81
Dorsal Striatum/Caudate	R	24	8	−2	704	4.04
Middle occipital gyrus	L	−38	−88	16	226	3.94
Anterior insula/superior temporal gyrus	L	−38	16	8	253	3.46

L, left; R, right; x, y, z, Montreal Neurological Institute (MNI) coordinates; Extent, number of voxels belonging to activated cluster; Z_max_, z-score at peak activated voxel within the cluster.

### General conflict processing

**Figure 2:**
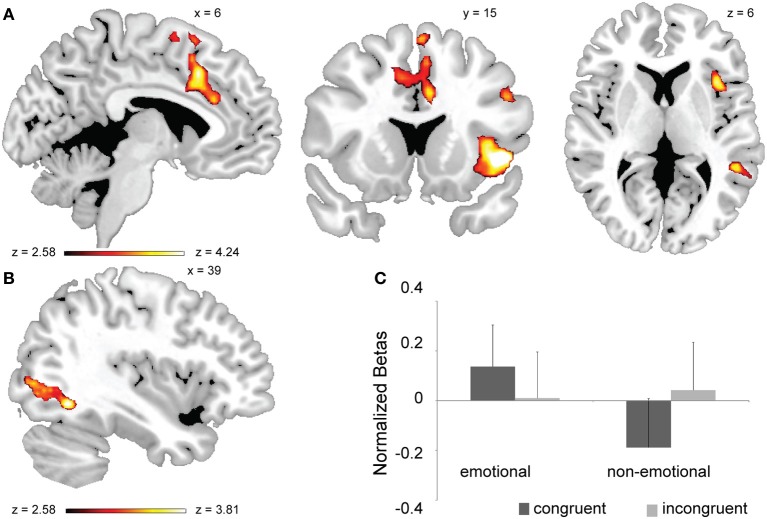
**Neural substrates of general conflict processing and its interaction with task-domain. (A)** Brain regions exhibiting a main effect of conflict (all incongruent > all congruent trials, whole-brain corrected at *p* < 0.05). **(B)** Brain regions displaying a conflict × task-domain interaction effect. **(C)** Mean activation estimates (+s.e.m.) for congruent and incongruent trials as a function of task-domain (non-emotional vs. emotional) are shown for the occipital activation cluster displayed in **(B)**.

To identify neural substrates of general conflict-related processing, we conducted a main effect contrast of the congruency factor (all incongruent > all congruent trials), collapsed across all other factors. We observed conflict-related activation in a large voxel-cluster covering bilateral dACC and stretching into the right pre-supplementary motor area (preSMA), as well as in additional clusters in the right inferior frontal gyrus and in right middle temporal gyrus (Figure 2A). In order to gauge whether conflict-driven activity in these regions was in any way modulated by task, we extracted mean cluster activation estimates and submitted them to congruency × task-domain interaction analyses. In none of the clusters was the congruency effect modulated by task (all *p* > 0.1), thus indicating a domain-general role in conflict-control processes for these regions.

We next carried out a whole-brain search for regions displaying a congruency × task-domain interaction effect. This type of activation pattern was observed in a cluster of voxels in the right middle occipital gyrus stretching into the right fusiform gyrus (Figure 2B). As gleaned from extracted mean beta values, this interaction was driven by greater conflict-related activity for the non-emotional task [incongruent > congruent, *F*_(1, 20)_ = 4.86, *p* < 0.05] than for the emotional one, where congruent trials evoked descriptively greater activity than incongruent ones [congruent > incongruent, *F*_(1, 20)_ = 3.61, *p* = 0.072] (Figure 2C). Thus, this region of extrastriate visual cortex appears to be selectively involved in conflict processing in the non-emotional domain. Note that there is a partial overlap between this cluster and the right FFA as defined in the independent localizer task, from which we report similar results below.

### Conflict processing modulated by global control context

**Figure 3:**
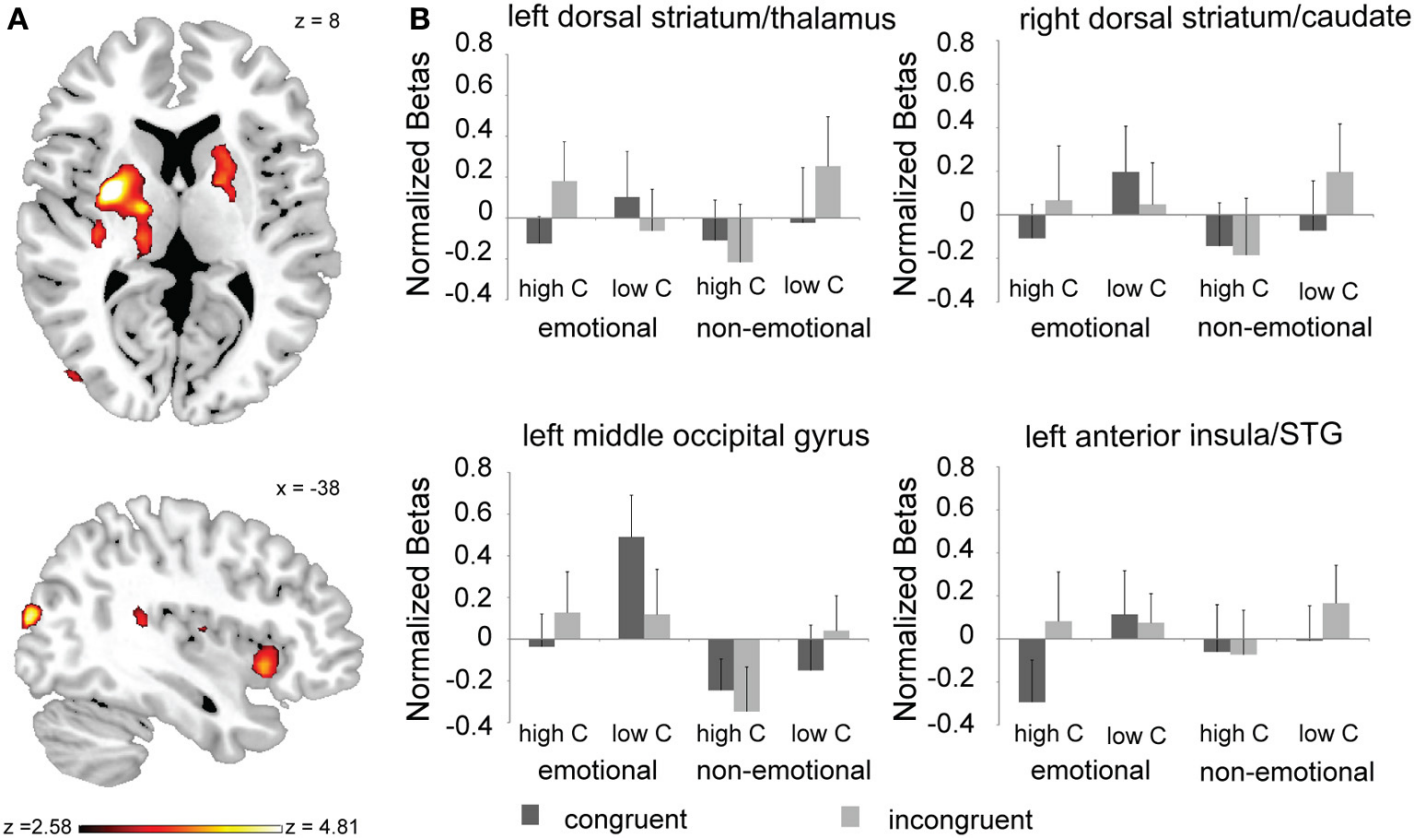
**Neural substrates conflict processing associated with the interaction between task-domain and global control context. (A)** Brain regions displaying a task-domain × proportion congruent × congruency interaction effect (whole-brain corrected at *p* < 0.05). **(B)** Mean activation estimates (+s.e.m.) for congruent and incongruent trials as a function of proportion congruency (high C, high proportion of congruent trials; low C, low proportion of congruent trials) and task-domain (emotional vs. non-emotional) are shown for the left dorsal striatum/thalamus and right dorsal striatum/caudate clusters displayed in **(A)** (top panel), and for the left insula/STG and left middle occipital gyrus activation clusters displayed in **(A)** (lower panel).

To identify brain regions where conflict processing was modulated by the global control context, we conducted both a 2-way congruency × proportion congruent interaction analysis (collapsing across the task-domain factor), as well as a 3-way congruency × proportion congruent × task-domain interaction analysis. We obtained no activation clusters passing whole-brain correction criteria in the congruency × proportion congruent analysis. However, a number of brain regions displayed differential modulation of conflict processing by global control context across task-domains. Specifically, 3-way interaction effects were observed in the dorsal striatum (DS), specifically the bilateral putamen and globus pallidus, with the left cluster stretching into the thalamus, and the right cluster extending into the caudate (Figure 3A). In addition to these subcortical activations, we also found 3-way interaction effects in left anterior insula cortex (stretching into the superior temporal gyrus) and in left middle occipital gyrus (Figure 3A).

Follow-up analyses showed that in each of these regions, the 3-way interaction effect was driven by the fact that global control context modulated the congruency effects to a larger degree in the emotional than in the non-emotional task-domain [left DS/thalamus: congruency × proportion congruency interaction for the emotional task, *F*_(1, 20)_ = 6.06, *p* < 0.05, non-emotional task, *F*_(1, 20)_ = 2.33, *p* > 0.1; right DS: congruency × proportion congruency interaction for the emotional task, *F*_(1, 20)_ = 4.3, *p* = 0.051, non-emotional task, *F*_(1, 20)_ = 2.8, *p* > 0.1; left insula: congruency × proportion congruency interaction for the emotional task, *F*_(1, 20)_ = 8.23, *p* < 0.01], non-emotional task [*F*_(1, 20)_ = 1.7, *p* > 0.1]; left middle occipital gyrus: congruency × proportion congruency interaction for the emotional task, *F*_(1, 20)_ = 16.26, *p* < 0.001, non-emotional task, [*F*_(1, 20)_ = 2.67, *p* > 0.1]. As can be seen in Figure 3B, in all of these regions there was greater activity for incongruent compared to congruent trials when the proportion of congruent trials was high/global control context low (and behavioral conflict effects are large) than when the proportion of congruent trials was low/global control context high (and behavioral conflict effects are small). Breaking up the subcortical clusters into peak activation estimates of their constituent nuclei did not reveal any findings that differed from the interaction patterns observed across the whole clusters. In sum, we found that the DS and left anterior insula displayed a functional dissociation in their response to conflict and global control context between emotional and non-emotional task domains.

### Conflict processing modulated by local control context

The effects of local control context on conflict processing (i.e., trial-by-trial congruency sequence effects) can be characterized in various ways. First, one can search for regions displaying a previous × current trial congruency interaction effect of the same (or inverse) direction observed in the behavioral conflict adaptation effect (Egner and Hirsch, 2005a). Alternatively, researchers have often focused on activation elicited by incongruent trials as a function of whether they have been preceded by a congruent trial or by an incongruent trial (Egner and Hirsch, 2005b; Etkin et al., 2006; Egner et al., 2008). Here, we applied both of these analytic strategies and tested the interaction of these contrasts with the task-domain factor. However, we detected no regions that passed the whole-brain correction statistical criterion for either analysis approach. In an additional, exploratory analysis we employed a rACC mask based on the results reported by Etkin et al. (2006), extracted mean beta estimates, and again conducted the above analyses (without whole-brain correction). However, even using this ROI-based approach, we detected no significant effects.

Given that we assessed this signature of local conflict-control in the context of a study designed to invoke systematic variation in global control context, it is possible these null-findings are attributable to the asymmetric distribution of congruent/incongruent trials. For instance, it is possible that trial-by-trial conflict-control would only be recruited under conditions where the global context would engender a low level of “proactive” control (i.e., when the proportion of congruent trials is high) (cf. Braver et al., 2007). To assess the possibility that the neural expression of local context effects were contingent on global context, we therefore conducted a 3-way previous trial congruency × current trial congruency × proportion congruent interaction analysis (collapsing across the task-domain factor). However, no clusters passing whole-brain correction were obtained for this analysis.

In additional exploratory analyses we investigated whether activations associated with local control could be detected when considering exclusively the high proportion congruent (i.e., low global control) condition. Here, we observed (under whole-brain correction) an interaction between previous and current trial congruency in a region of the ACC/dmPFC (peak effect at *x* = 6, *y* = 44, *z* = 26, cluster size = 280 voxels) slightly dorsal to the rostral ACC region identified as mediating local emotional conflict-control in prior studies (e.g., Etkin et al., 2006: peak effect at *x* = −10, *y* = 48, *z* = 0; Egner et al., 2008: peak effect at *x* = −12, *y* = 44, *z* = −2). When comparing the previous by current trial interaction in this cluster's activation between emotional and non-emotional domains, we observed no interaction effect, however, as the effects of local control were observed to a similar degree in both tasks [non-emotional task, *F*_(1, 20)_ = 8.23, *p* = 0.04; emotional task, *F*_(1, 20)_ = 6.70, *p* = 0.017]. For both tasks, this local control context effect in the high proportion congruent condition reflected a relative increase in incongruent > congruent trial activity following an incongruent compared to a congruent trial, an activation signature that tracks the assumed level of local conflict-control (Egner and Hirsch, 2005a,b). Thus, this rostral ACC region showed similar local control effects to those reported previously for a slightly more ventral rACC focus (Etkin et al., 2006; Egner et al., 2008), but this effect was only observed in the high proportion congruent blocks, and it was not selective to the emotional domain. In any case, these findings should be interpreted with caution, however, as they stem from *post-hoc* tests that were not motivated by a significant higher-order interaction effect at whole-brain corrected statistical threshold.

### Regions of interest analyses

**Figure 4:**
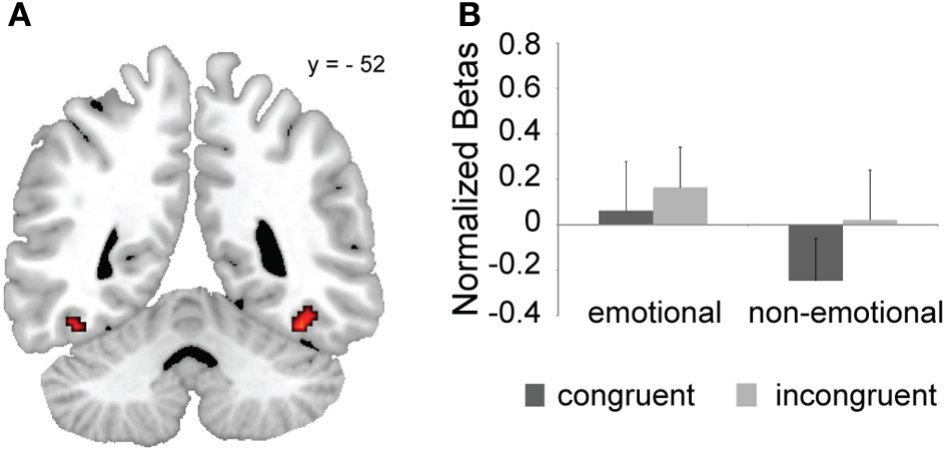
**FFA ROI results. (A)** Group FFA ROIs based on an independent localizer scan (uncorrected *p* < 0.005 and cluster-size = 20). **(B)** Congruent and incongruent mean activation estimates (+s.e.m.) as a function of task-domain are shown averaged across the left and right FFA ROIs from panel **(A)**.

We carried out an independent localizer scan to define face-sensitive ROIs, in particular the FFA and amygdala, in order to test whether the processing of face stimuli was modulated by global or local domain-dependent conflict-control processes. Figure 4A displays the FFA group activation map. We first tested whether any of our effects of interest were modulated by FFA laterality. Since we did not find any significant interaction involving this factor, we collapsed across left and right FFAs and performed the same analyses that we had previously conducted at the whole-brain level. We found a task × congruency interaction, *F*_(1, 20)_ = 5.69, *p* < 0.05, with significant incongruent > congruent activation differences for the non-emotional task [*F*_(1, 20)_ = 11.95, *p* < 0.005], but not for the emotional task [n.s. *F*_(1, 20)_ = 2.57, *p* > 0.1] (Figure 4B). Thus, as already reported for a partly overlapping cluster in the right occipital cortex (Figures 2B,C), the FFA in general was susceptible to conflicting distracters in the non-emotional domain, but not in the emotional domain (cf. Egner et al., 2008). No other significant effects were observed.

The localizer task also fashioned us with functional amygdala ROIs. Since these face-sensitive activation clusters extended beyond the anatomical confines of the amygdala proper, we masked the functional ROIs with an anatomically defined amygdala ROI (see Methods). Mean activation estimates of the resultant clusters were submitted to the same analyses as the FFA ROIs. Once more, we did not find any interaction with laterality (*F* < 1) and therefore collapsed the analysis across this factor. However, for the amygdala, we did not detect any main effect of congruency or interactions between congruency and any of the other factors.

## Discussion

In this study, we examined the possibility that there are dissociable neural mechanisms involved in the modulation of emotional vs. non-emotional conflict-control processes by global control context, by varying the source of conflict, between emotional and non-emotional, and assessing behavioral and neural effects of conflict and its modulation by global control context (in a proportion congruent manipulation). In addition, we also explored the impact of local control context (by assessing congruency sequence effects) as a function of global context. At the behavioral level, we observed effects of global control context, with larger congruency effects when the proportion of congruent trials was high than when it was low. Similarly, we also observed effects of local control context, as reflected in congruency sequence effects. Neither of these effects was reliably modulated by task-domain, and global and local control context effects had additive (non-interactive) effects on behavior. The latter finding suggests that global and local control effects may rely on distinct mechanisms, an idea that finds support in previous behavioral studies (Funes et al., 2010; Torres-Quesada et al., 2013). In fact, in the present study we also observed neural signatures of proportion congruent effects in the absence of corresponding congruency sequence effects (e.g., in the DS and insula, see below), thus adding more weight to these prior findings. Moreover, these behavioral data provided a solid basis for assessing whether there were distinct neural substrates involved in the contextual modulation of conflict processing in the emotional vs. non-emotional domain.

At the neural level, we obtained three main results, which we will discuss in turn: first, we observed domain-general conflict-related activation in a set of regions prominently including the dorsal ACC and preSMA; second, we found a conflict effect specific to the non-emotional task in lateral and ventral visual cortex, including the fusiform gyrus/FFA; third, and most importantly, we documented the existence of dissociable, domain-specific neural substrates of conflict-control processes, with particularly the DS and anterior insula tracking conflict as modulated by global control context in the emotional but not in the non-emotional task. Finally, it is important to note that all the activations described above tracked behavioral conflict scores, i.e., more neural activity was observed when interference was high than when it was low, as would be expected from brain regions that are involved either in the genesis or the evaluation (e.g., detection/monitoring) of conflict. Moreover, recall that when we discuss effects of global control context, we are not referring to sustained (block-wise) activations, but rather to transient (event-related) signals whose amplitudes were modulated as a function of control context (i.e., the proportion congruent manipulation).

As noted above, we observed generic (main effect of incongruent > congruent trials) and domain-general conflict-related activation in a large voxel-cluster covering dACC and stretching into the right preSMA, as well as in additional clusters in the right inferior frontal gyrus and in right middle temporal gyrus. Especially the former three are areas that have been extensively reported in the conflict-control literature (e.g., Carter et al., 1998; Botvinick et al., 1999; Casey et al., 2000; MacDonald et al., 2000; Kerns et al., 2004; Egner, 2011). Moreover, the overlap of emotional and non-emotional conflict signals in the dACC we observed in the present study in the context of a proportion congruency manipulation replicates a previous finding of overlapping emotional and non-emotional conflict signals in the context of local control (congruency sequence) effects (Egner et al., 2008). The current data therefore reinforce the idea that the dACC plays a central role in conflict processing, perhaps reflecting in particular the common response conflict component of the non-emotional and emotional tasks we employed.

We next tested for brain regions where the effect of conflict was modulated by task-domain. We found domain-specific activations in the right middle occipital gyrus stretching into the right fusiform gyrus, in that these regions were susceptible to non-emotional but not to emotional congruency effects. We also observed this same type of domain-specificity for the FFA, as defined by an independent localizer task. The finding that the FFA appears to be exclusively involved in conflict processing in the non-emotional task-domain again represents a replication of prior work that focused on congruency sequence effects (Egner et al., 2008). Together, these studies suggest that the generation and regulation of conflict involving face stimuli recruits the FFA and nearby extrastriate visual regions if the conflict is non-emotional in nature, but depends on other (particularly subcortical) regions when emotional processing is at the root of conflict generation (Etkin et al., 2006, 2010; Egner et al., 2008).

The main goal of the current study was to probe whether there are dissociable neural mechanisms for of global control context modulation of conflict-control processes in emotional and non-emotional domains contexts. This possibility was raised by a recent study of proportion congruent effects in an emotional conflict task (Krug and Carter, 2012), but that study lacked a non-emotional comparison condition. Our fMRI data clearly support this proposal. The modulation of conflict by global control context in the emotional, but not in the non-emotional domain, was associated with activity in the DS, specifically, bilateral putamen and globus pallidus, with the left cluster stretching into the thalamus, and the right cluster extending into the caudate; left anterior insula cortex (stretching into the superior temporal gyrus); and left middle occipital gyrus. In these regions there was greater activity for incongruent compared to congruent trials when the proportion of congruent trials was high/global control context low (and behavioral conflict effects are large) than when the proportion of congruent trials was low/global control context high (and behavioral conflict effects are small). This response profile has previously been interpreted as reflecting conflict processing as modulated by sustained or proactive control processes (e.g., Carter et al., 2000).

What exactly might be the role of the DS in the emotional proportion congruent effect? Possible candidates include the DS' putative involvement in inhibitory control functions (Rubia et al., 2006; Ali et al., 2010; Beste et al., 2012); perhaps relatedly, its implication in overriding more habitual in favor of less habitual, “goal-directed” responses (Tanaka et al., 2008; Tricomi et al., 2009; de Wit et al., 2012); and finally, its well-established role in associative learning processes (Buch et al., 2006; Zedhoka et al., 2006; Hubert et al., 2007; Brovelli et al., 2011). In principle, any or all of these putative DS functions could contribute to our findings, though some appear more feasible than others when it comes to accounting for the fact that the DS involvement was specific to the emotional task-domain.

Firstly, the emotional face-word Stroop task may well-require inhibitory control and certainly involves the override of a more habitual (word-reading) response with a less habitual (facial affect labeling) one. Intuitively, it is not obvious though why these requirements would be more pronounced in the emotional than in the non-emotional task version, but there is actually prior evidence suggesting that the resolution of emotional conflict may be more reliant on inhibitory mechanisms than the resolution of non-emotional conflict, at least in the context of the present tasks. Specifically, while local control context effects in the non-emotional version of this task have been shown to involve excitatory biasing in favor of processing task-relevant face stimuli (Egner and Hirsch, 2005b), the corresponding effects in the emotional version have instead been found to involve the inhibition of affective responses to the emotional distracter stimuli (Etkin et al., 2006). The latter process has previously been associated with the attenuation of amygdala responses by input from the rACC (Etkin et al., 2006; Egner et al., 2008), but in the case of longer-term, global control context effects, it may well-involve a different pathway that includes the DS.

Secondly, global control context effects as assessed in a proportion congruency manipulation are thought to be an expression of associative learning (e.g., Botvinick et al., 2001; Verguts and Notebaert, 2008), e.g., the association of a temporal context (a block of trials) with a high frequency of conflict, resulting in strategic adjustments of attention (cf. King et al., 2012). Therefore, the activations found in the DS could be related to the acquisition of these context-control associations. It is not clear though if and why this expression of associative learning would be exclusive to the proportion congruency effect in the emotional domain. To explore the possibility of differential learning effects in the two tasks further, we conducted a *post-hoc* analysis on proportion congruent effects (context × congruency interaction) as a function of time within each block (“learning”: first half vs. second half of block) and task. While we did not obtain a significant 4-way interaction effect indicative of differential learning between tasks (*P* > 0.1), the numerical values are suggestive of the possibility that learning within blocks of trials was more pronounced in the emotional task (proportion congruent effect in first half: 3 ms; second half: 37 ms) than in the non-emotional task (proportion congruent effect in first half: 11 ms; second half: 15 ms). Thus, it is possible that learning mechanisms involving the DS may be preferentially engaged by emotional stimuli, perhaps due to their intrinsic value or affective salience. In line with this possibility, namely that the DS may display some selective susceptibility to emotionally salient stimuli, some recent studies have indeed observed dorsal striatal responses specific to emotional distracters and clinical status, distinguishing between bipolar and unipolar depressed individuals (Bertocci et al., 2012) and biploar patients and healthy controls (Mullin et al., 2012). Ultimately, however, the precise functional role the DS plays in the modulation of conflict responses by global control context still needs to be studied further.

Another area found to be specifically associated with the modulation of emotional conflict by global control context is the (left anterior) insula, a paralimbic cortical region that is considered to be a core component of affective processing systems in the human brain (e.g., Kober et al., 2008; Lindquist et al., 2012). Context-modulated conflict effects in the insula have in fact been reported previously, both in non-emotional (Carter et al., 2000; Grandjean et al., 2012; Wilk et al., 2012) and emotional task contexts (Krug and Carter, 2012). Importantly, the present study, being the first to directly contrast global control context effects between emotional and non-emotional tasks, was able to test whether this region shows preferential conflict-tracking responses in either context. Our results document that phasic, control-modulated conflict signals in the left anterior insula were in fact specific to the emotional task context, suggesting a domain-specific (or at least preferential) role in emotional conflict-tracking for this region. This finding fits well with a recent study by Chechko et al. (2012), who observed stronger left anterior insula responses to emotional than non-emotional conflict in a face-word Stroop task analogous to our own (but without a proportion congruent manipulation), thus suggesting a robust preferential involvement of the insula in emotional conflict processing.

One possible functional role of the insula in this regard is highlighted by a recent study suggesting that this region might integrate signals of cognitive demand with affective stimulus salience (Gu et al., 2013). Along similar lines, an intriguing speculative interpretation of the insula's role in the present study could rely on its involvement in a putative “salience network” (Seeley et al., 2007) that facilitates the detection of important environmental stimuli, and initiates attentional control signals in turn (e.g., Menon and Uddin, 2010). Specifically, the insula may be involved in detecting emotional conflict (and emotionally salient stimuli in general, see Dolcos and McCarthy, 2006) and interact with the DS in triggering control processes geared at overcoming that conflict. Note that an alternative, task-difficulty based explanation for selective conflict-related effects in these regions during the emotional task based on task difficulty is not supported by our data. Specifically, we observed no main effect of task on behavior, nor any significant interactions involving the task-domain factor, thus suggesting that the two task versions were of comparable difficulty.

Finally, it is important to note that the analysis of local control context (congruency sequence) effects in the emotional domain did not replicate previous findings in the amygdala (Etkin et al., 2006, 2010) or in the rACC (Etkin et al., 2006; Egner et al., 2008; Maier and di Pellegrino, 2012). The most likely reason for these unexpected null-results is the very fact that the current protocol involved a manipulation of global control context (involving a skewed distribution of congruent/incongruent stimuli), which appears to change the dynamics of the local control context effects in comparison to conditions where the incidence of congruent and incongruent trials is balanced (Etkin et al., 2006, 2010; Egner et al., 2008). In line with this idea, in some exploratory *post-hoc* analyses we observed a (more dorsal) rACC focus displaying local control effects (akin to prior studies), and this was the case for the high proportion congruent (low global control context) condition only. However, these effects were also domain-general. Overall, the present data cast doubt on whether the rACC plays its well-established role in emotional conflict adaptation in the context of a simultaneous manipulation of global control context.

The general lack of any task-domain effects in the amygdala represents a second unexpected null-finding, given the well-established specialization of this region for detecting emotionally salient stimuli (e.g., LeDoux, 1996) as well as previous findings in emotional conflict-control experiments (Etkin et al., 2006; Egner et al., 2008). One feasible explanation is that both of the current tasks involved the continuous differentiation of facial features (to determine either gender or affect), which, given the amygdala's general responsiveness to face stimuli (e.g., Ishai, 2008), may have created a ceiling effect that did not allow us to detect a significant additional enhancement of amygdala activity in the emotional compared to the non-emotional task context.

Previous imaging studies in the clinical domain that investigated emotional conflict processing have focused in particular on the modulation of conflict by local control context in patients with mood disorders. For instance, Etkin and colleagues observed diminished behavioral effects and neural engagement of control-related rACC-amygdala circuitry (cf. Etkin et al., 2006; Egner et al., 2008) in response to local control context in an emotional conflict task in patients with generalized anxiety disorder (Etkin et al., 2010) and a compensatory shift in neural engagement to lateral PFC in patients with major depressive disorder (Etkin and Schatzberg, 2011). These findings suggest that emotional conflict regulation driven by local context is impaired in mood disorders, but to our knowledge no previous study involving these clinical groups has assessed the modulation of emotional conflict responses by global control context. Interestingly, neural responses to negative emotional stimuli in major depressive disorder are in fact known to be abnormal in both the insula and the dorsal striatum (e.g., Hamilton et al., 2012), the two key regions implicated in global control context modulation of emotional conflict responses in the present data set. Thus, it would be of particular interest to test whether these neural abnormalities would translate into impaired modulation of emotional conflict processing by global context, which could be assessed by applying the present experimental protocol to these patients.

## Conclusions

In summary, we present a novel finding of a partial dissociation between neural conflict processing, as modulated by global control context, in the emotional as compared to the non-emotional domain. This partial dissociation mirrors that previously observed for mechanisms mediating local control context effects in the emotional vs. non-emotional tasks (Etkin et al., 2006; Egner et al., 2008), but it involved distinct neural substrates. Most notably, the dorsal striatum and anterior insula appears to play a selective role in tracking emotional conflict and its' modulation by global control context in the emotional domain.

### Conflict of interest statement

The authors declare that the research was conducted in the absence of any commercial or financial relationships that could be construed as a potential conflict of interest.
